# Heat and mass transfer analysis in unsteady flow of tangent hyperbolic nanofluid over a moving wedge with buoyancy and dissipation effects

**DOI:** 10.1016/j.heliyon.2020.e03776

**Published:** 2020-04-16

**Authors:** Tesfaye Kebede, Eshetu Haile, Gurju Awgichew, Tadesse Walelign

**Affiliations:** Department of Mathematics, Bahir Dar University, Po. Box 79, Bahir Dar, Ethiopia

**Keywords:** Mechanical engineering, Theoretical fluid dynamics, Computational fluid dynamics, Nanofluidics, Heat transfer, Mass transfer, Magnetohydrodynamics, Mathematical physics, Tangent hyperbolic nanofluid, Heat and mass transfer, Moving wedge, Homotopy analysis method

## Abstract

In this study, a convective heat and mass transfer phenomena in a time-dependent boundary layer flow of tangent hyperbolic nanofluid over a permeable stretching wedge has been examined with respect to some pertinent thermo-physical parameters. Convenient similarity transformation is used to reformulate the dimensional partial differential equations into dimensionless system of ordinary differential equations. The reduced set of equations is solved by the homotopy analysis method implemented in Mathematica environment. The effects of the relevant parameters on velocity, temperature and concentration profiles were examined in detail. The impacts of the parameters on the rates of momentum, heat and mass transfer are also analyzed quantitatively in terms of the wall friction coefficient, local Nusselt number and Sherwood number, respectively. Analysis of the results reveals that the increase in the buoyancy ratio parameter facilitates the flow velocity and the increase in the dissipation parameter maximizes the temperature distribution and nanoparticle concentration near the surface of the wedge. Moreover, the analytic approximations obtained by implementing the homotopy analysis method are found in excellent agreement with some previously published results.

## 1 Introduction

In manufacturing industries, the rate of heat transfer has significant effects on cost of production and quality of products. Also, effective heating and/or cooling are top technical challenges facing high-tech industries, automobile engines, transformers and many other technological devices. In order to enhance the thermal conductivity of traditional fluids such as engine oils, water and air, Choi [1] introduced the concept of nanofluids, which refers to innovative fluids comprising of base liquids with uniform and stable suspension of nano-sized particles. A comprehensive analysis for the reason behind the extraordinary thermal conductivity of nanofluids was reported by Buongiorno [2]. With this understanding, a number of investigations have been conducted to outline the boundary layer flow of nanofluids with heat and mass transfer phenomena. For instance, the recent studies of Haile and Awgichew [3] revealed that the increase in hydrodynamic slip reduces the velocity of the nanofluid; but it enhances the temperature and nanoparticle volume fraction. Some other studies on boundary layer flow of nanofluids are also indicated in the cited articles [4], [5], [6], [7], [8], [9], [10].

On the other hand, many of the fluids processed in manufacturing industries including paints, lubricants, detergents, polymeric liquids, molten plastics and food staffs do not obey the Newton's law of viscosity. That is, the relation between the shear stress and the shear rate in such fluids is not linear and their viscosities vary differently in response to shear stress. These features of the fluids add more complexities in the resulting equations and the classical Newtonian models are not appropriate to describe and predict many critical behaviors of such fluids. Consequently, several constitutive equations are used to describe the behavior of non-Newtonian fluids. The various constitutive models for the non-Newtonian fluids are available in [11]. Tangent hyperbolic model, first introduced by Pop and Ingham [12], is one of the non-Newtonian fluid models used to understand and predict diverse flow properties of industrial fluids like paints, nail polish, ketchup, whipped cream, etc. Owing to its remarkable applications in modern industries, several researchers have been considering tangent hyperbolic model to examine the flow characteristics of many industrial fluids under different thermo-physical conditions. For example, Prabhakar et al. [4], Ibrahim [5], Saidulu et al. [6], Mahdy and Hoshoudy [7] and Shahzad et al. [8] reported their numerical studies on magnetohydrodynamic flow of tangent hyperbolic nanofluid over stretching sheets subjected to different initial and boundary conditions.

Also, flows over wedge shaped surfaces are important area of research as such flows encountered in several scientific and industrial investigations including hydrodynamics, aerodynamics, magnetohydrodynamics, analysis of geothermal systems, thermal insulation, crude oil exploration and extraction, heat exchangers, storage of nuclear wastes, etc. Falkner and Skan explored the flow over a static wedge in the beginning of 1930's. Later in 1937, Hartree [13] inspected the dependence of solutions on wedge angle parameter. A number of studies were devoted to analyze the impact of different parameters on the wedge flow and heat transfer characteristics of nanofluids. For instance, Ullah et al. [14], Hashim et al. [15] and Jyothi et al. [16] considered flow phenomena and heat transfer properties of certain non-Newtonian fluids over wedge-shaped surfaces.

However, to the best of the authors' knowledge, only few studies on a time dependent magnetohydrodynamic flow of tangent hyperbolic nanofluid past a stretching wedge are reported in open literature. For instance, Mahdy and Chamkha [9] utilized the Keller box method to give numerical approximations for the boundary layer flow of two-phase tangent hyperbolic nanofluids over a stretched wedge moving in a porous medium. Also Atif et al. [10] employed the shooting technique to analyze heat and mass transfer of tangent hyperbolic nanofluid past a wedge. However, both the studies did not examine the effects of certain relevant parameters such as permeability of the wall, buoyancy force, viscous dissipation, Joule heating and heat source. Thus, motivated by the aforementioned studies, we made an effort to examine the effects of the indicated parameters. On the other hand, a reliable semi-analytic and semi-numerical method, namely the homotopy analysis method, is used to give analytic approximations for the solution of the resulting nonlinear equations. Convergence of the method is ensured by plotting both the h-curves and graph of the average squared residual error. In order to further validate the accuracy of our results, comparisons are made between certain results of the present study and some previously published studies under common assumptions; and they are found in excellent agreement.

## 2 Model assumptions and mathematical formulations

**Figure 1:**
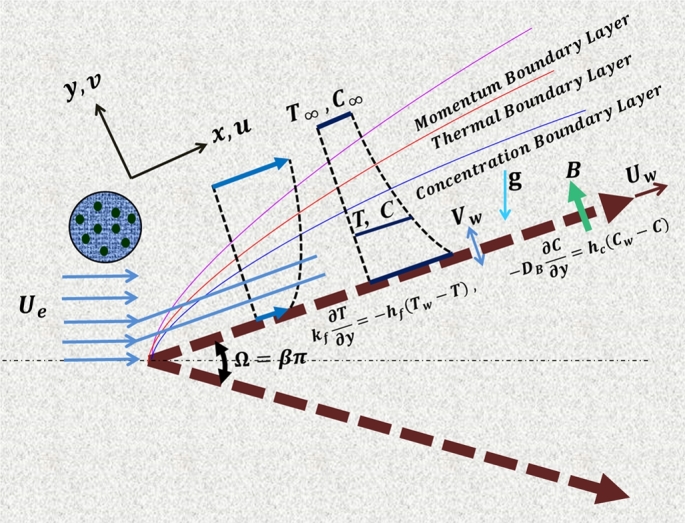
Flow configuration and coordinate system.

Consider a two-dimensional laminar flow of an incompressible tangent hyperbolic nanofluid past the surface of a permeable wedge embedded in a porous medium. The Cartesian coordinate system (x,y) is chosen in such a way that the origin is fixed at the apex of the wedge, the x-axis is directed along the wedge surface and the y-axis is normal to the wedge surface as illustrated in Fig. 1. Assume that the flow is induced by stretching of the wedge with wall velocity $\;U_{w}(x,t)=\frac{ax^{m}}{1-ct}$ and external flow of the fluid with stream velocity $\;U_{e}(x,t)=\frac{bx^{m}}{1-ct}$ subjected to a magnetic field $\text{B}=(0,B_{0})$ acting normal to the wedge surface. Here, *t* is the time variable; a,b,m and *c* are constants such that a>0 and a<0 denote stretching and shrinking rates of the wedge, respectively; *m* is the Falkner-Skan power-law constant defined as $m=\frac{\beta }{2-\beta }$ with 0≤m≤1 and *β* being the Hartree pressure gradient that can also be described in terms of the total wedge angle Ω by $\beta =\frac{\Omega }{\pi }$. Assume the surface temperature $T_{w}$ and concentration $C_{w}$ of the wedge vary in power-law forms as $T_{w}=T_{\infty }+\frac{bx^{m}}{1-ct}$ and $C_{w}=C_{\infty }+\frac{bx^{m}}{1-ct}$, where $T_{\infty }$ and $C_{\infty }$ are the constant values of the ambient temperature and concentration, respectively.

With all the above assumptions, the conservation laws governing the flow phenomena are expressed by the continuity equation, momentum equation, energy equation and concentration equation, given respectively as follows:

(1) $$\frac{\partial u}{\partial x}+\frac{\partial v}{\partial y}=0,$$

(2) $$\frac{\partial u}{\partial t}+u\frac{\partial u}{\partial x}+v\frac{\partial u}{\partial y}=\frac{\partial U_{e}}{\partial t}+U_{e}\frac{\partial U_{e}}{\partial x}+\upsilon \left[(1-n)+\sqrt{2}n\Gamma \frac{\partial u}{\partial y}\right]\frac{\partial^{2}u}{\partial y^{2}}\;\;-\left(\frac{\sigma B_{0}^{2}}{\rho_{f}}+\frac{\mu }{K_{1}}\right)(u-U_{e})+g[\beta_{T}(T-T_{\infty })-\beta_{c}(C-C_{\infty })]sin\left(\frac{\Omega }{2}\right),$$

(3) $$\frac{\partial T}{\partial t}+u\frac{\partial T}{\partial x}+v\frac{\partial T}{\partial y}=\alpha \frac{\partial^{2}T}{\partial y^{2}}+\tau \left[D_{B}\frac{\partial C}{\partial y}\frac{\partial T}{\partial y}+\frac{D_{T}}{T_{\infty }}{\left(\frac{\partial T}{\partial y}\right)}^{2}\right]\;+\frac{16\sigma^{⁎}T_{\infty }^{3}}{3{\left(\rho C_{p}\right)}_{f}k^{⁎}}\frac{\partial^{2}T}{\partial y^{2}}+\frac{\sigma B_{0}^{2}}{\rho c_{p}}{\left(u-U_{e}\right)}^{2}+\frac{\upsilon }{c_{p}}\left[(1-n){\left(\frac{\partial u}{\partial y}\right)}^{2}+\frac{n\Gamma }{\sqrt{2}}{\left(\frac{\partial u}{\partial y}\right)}^{3}\right]\;+\frac{Q_{0}}{{\left(\rho C_{p}\right)}_{f}}(T-T_{\infty }),$$

(4) $$\frac{\partial C}{\partial t}+u\frac{\partial C}{\partial x}+v\frac{\partial C}{\partial y}=D_{B}\frac{\partial^{2}C}{\partial y^{2}}+\frac{D_{T}}{T_{\infty }}\frac{\partial^{2}T}{\partial y^{2}},$$

where (u,v) are components of the fluid velocity along the x-axis and y-axis, respectively; *T* and *C* are the dimensional temperature and concentration of the nanofluid in the boundary layer region; *ρ* and *μ* are the density and dynamic viscosity of the nanofluid, respectively; $\upsilon =\frac{\mu }{\rho }$ is kinematics viscosity; *n* is the power law fluid viscosity index representing the flow behavior of the tangent hyperbolic fluid. The quantities $\Gamma ,g,K_{1},\beta_{T}$ and $\beta_{C}$ denote time-dependent material constant, the magnitude of gravitational acceleration, permeability of the porous medium and the volumetric thermal and concentration expansion coefficients, respectively. The term $\alpha =\frac{k_{f}}{{\left(\rho C_{p}\right)}_{f}}$ is the effective thermal diffusivity, where $k_{f}$ is thermal conductivity of the nanofluid; $\tau =\frac{{\left(\rho C_{p}\right)}_{p}}{{\left(\rho C_{p}\right)}_{f}}$ is the ratio of effective heat capacities of nanoparticle and the base fluid with $C_{p}$ as the specific heat at constant pressure; $D_{B}$ and $D_{T}$ are respectively the Brownian and thermophoresis diffusion coefficients; $k^{⁎}$ and $\sigma^{⁎}$ are the mean absorption and the Stefan-Boltzmann constants, respectively; and the coefficient $Q_{0}$ represents the heat generation (when $Q_{0}>0$) or the heat absorption (when $Q_{0}<0$).

The boundary conditions at the surface of the wedge and far from it are taken as follows

(5) $$u=U_{w}(x,t),\;\;\;v=V_{w}(t),\;\;\;-k_{f}\frac{\partial T}{\partial y}=h_{f}(T_{w}-T),-D_{B}\frac{\partial C}{\partial y}=h_{c}(C_{w}-C)\;\;\;\text{at}\;\;y=0,$$

(6) $$u\to U_{e}(x,t)=\frac{bx^{m}}{1-ct},\;\;\;T\to T_{\infty },\;\;\;C\to C_{\infty }\;\;\;\;\text{as}\;\;\;y\to \infty$$

where $V_{w}=\frac{V_{0}}{\sqrt{1-ct}}$ is the transpiration velocity representing the mass transmission at the surface of the stretching wedge with $V_{0}$ as the constant value of velocity; $h_{f}$ and $h_{c}$ are respectively the convective heat and mass transfer coefficients.

In order to reformulate the system of partial differential equations into simple set of ordinary differential equations, the following similarity transformations are used:

(7) $$\eta =y\sqrt{\frac{(1+m)U_{e}}{2\upsilon (1-ct)}},\;\;\;\psi (x,y,t)=\sqrt{\frac{2\upsilon xU_{e}}{1+m}}f(\eta ),\;\;\theta (\eta )=\frac{T-T_{\infty }}{T_{w}-T_{\infty }}\;\;\;\text{and}\;\varphi (\eta )=\frac{C-C_{\infty }}{C_{w}-C_{\infty }},$$

where *η* stands for the dimensionless similarity variable; *ψ* is the stream function having the properties $u=\frac{\partial \psi }{\partial y}$ and $v=-\frac{\partial \psi }{\partial x}$; f(η),θ(η) and φ(η) denote the dimensionless stream function, temperature and nanoparticle volume fraction, respectively.

Up on computing the required quantities and their partial derivatives and substituting the values into the governing equations, the continuity equation (1) is satisfied identically and the Eqs. (2)-(4) are reduced to the following dimensionless set of ordinary differential equations:

(8) $$\left[(1-n)+nWe\sqrt{1+m}f^{\prime \prime }\right]f^{\prime \prime \prime }+ff^{\prime \prime }+\frac{2m}{1+m}(1-f^{\prime \;2})\;+\frac{A}{1+m}(2-2f^{\prime }-\eta f^{\prime \prime })+\frac{2m}{1+m}(M+Da)(1-f^{\prime })\;+(Gr\theta +Gc\varphi )sin\left(\frac{\Omega }{2}\right)=0,$$

(9) $$\frac{1}{Pr}\left(1+\frac{4}{3}Rd\right)\theta^{\prime \prime }+N_{b}\theta^{\prime }\varphi^{\prime }+N_{t}\theta^{\prime \;2}+f\theta^{\prime }-\frac{2m}{1+m}f^{\prime }\theta -\frac{A}{1+m}(\eta \theta^{\prime }+2\theta )+\frac{2m}{1+m}MEc\left(f^{\prime \;2}-2f^{\prime }+1\right)+Ec\left[(1-n)f^{\prime \prime \;2}+nWef^{\prime \prime \;3}\right]+\frac{2m}{1+m}Q=0,$$

(10) $$\varphi^{\prime \prime }+PrLe\left(f\theta^{\prime }-\frac{2m}{1+m}f^{\prime }\varphi -\frac{A}{1+m}(\eta \varphi^{\prime }+2\varphi )\right)+\frac{N_{t}}{N_{b}}\theta^{\prime \prime }=0,$$

where the prime ′ indicates differentiation with respect to *η*; $Ec=\frac{U_{w}^{2}}{{\left(C_{p}\right)}_{f}(T_{w}-T_{\infty })}$ is the Eckert number representing dissipation effects; $Da=\frac{\upsilon x}{K_{1}U_{e}}$ is the Darcy number for porosity of the medium; $Gr=\frac{g\beta_{T}(T_{w}-T_{\infty })x}{U_{\infty }^{2}}$ and $Gc=\frac{g\beta_{c}(C_{w}-C_{\infty })x}{U_{\infty }^{2}}$ are the thermal and mass buoyancy parameters, respectively. The quantities $N_{b}=\frac{\tau D_{B}(C_{w}-C_{\infty })}{\upsilon }$ and $N_{t}=\frac{\tau D_{T}(T_{w}-T_{\infty })}{\upsilon T_{\infty }}$ correspond to the Brownian motion and the thermophoresis parameters, respectively; $Q=\frac{xQ_{0}}{{\left(\rho C_{p}\right)}_{f}U_{w}}$ stands for the heat generation (when Q>0) or heat absorption (when Q<0). In addition, the Weissenberg number *We*, unsteadiness parameter *A*, magnetic field parameter *M*, Prandtl number *Pr*, thermal radiation parameter *Rd* and Schmidt number *Sc* are defined respectively as $We=\frac{\Gamma U_{e}^{3/2}}{\sqrt{\upsilon }x},\;\;\;A=\frac{c}{bx^{m-1}},\;\;\;M=\frac{\sigma B_{0}^{2}x}{\rho U_{e}},\;\;P_{r}=\frac{\upsilon }{\alpha },\;\;R_{d}=\frac{4\sigma^{⁎}T_{\infty }^{3}}{k_{f}k^{⁎}}$ and $Le=\frac{\alpha }{D_{B}}$.

It is important to note that the mathematical model will reduce to the Newtonian viscous flow model as n→1 and We→0. Also employing the similarity transformation in Eq. (7), the boundary conditions in Eqs. (5) and (6) are simplified as

(11) $$f(0)=S,\;\;\;\;f^{\prime }(0)=\delta ,\;\;\;\;\theta^{\prime }(0)+Bi_{1}[1-\theta (0)]=0,\varphi^{\prime }(0)+Bi_{2}[1-\varphi (0)]=0,$$

(12) $$f^{\prime }(\eta )\to 1,\;\;\;\;\theta (\eta )\to 0,\;\;\;\;\varphi (\eta )\to 0\;\;\;\;\;\text{as}\;\eta \to \infty ,$$

where the parameter $S=\frac{V_{0}}{\sqrt{a\upsilon }}$ represents suction (when S<0) and injection (when S>0); $\delta =\frac{U_{e}}{U_{w}}$ denotes the velocity ratio parameter; the parameters $Bi_{1}=\frac{h_{f}}{k_{f}}\sqrt{\frac{2\upsilon }{U_{e}(m+1)}}$ and $Bi_{2}=\frac{h_{s}}{Da}\sqrt{\frac{2\upsilon }{U_{e}(m+1)}}$ are the Biot numbers for heat and mass diffusion, respectively.

From the practical point of view, it is worth predicting the behavior of the three most useful physical quantities, namely the wall friction $C_{f}$, the local Nusselt number $Nu_{x}$ and Sherwood number $Sh_{x}$ which are given by:

$$C_{f}=\frac{\tau_{w}}{\rho_{f}U_{e}^{2}},\;\;\;Nu_{x}=\frac{xq_{w}}{\alpha (T_{w}-T_{\infty })}\;\;\;\text{and}\;\;\;Sh_{x}=\frac{xq_{m}}{D_{B}(C_{w}-C_{\infty })}$$

where $\tau_{w}=\mu {\left[(1-n)\frac{\partial u}{\partial y}+\frac{n\Gamma }{\sqrt{2}}{\left(\frac{\partial u}{\partial y}\right)}^{2}\right]}_{y=0}$, $q_{w}=-{\left[\left(\alpha +\frac{16\sigma^{⁎}T_{\infty }^{3}}{3{\left(\rho C_{p}\right)}_{f}k^{⁎}}\right)\frac{\partial T}{\partial y}\right]}_{y=0}$ and $q_{m}=-D_{B}{\left(\frac{\partial C}{\partial y}\right)}_{y=0}$ are respectively the surface shear stress, surface heat flux and mass flux. By substitution and using the similarity transformations, we obtain the following relations

$$\sqrt{Re}C_{f}=\left[\sqrt{1+m}(1-n)+\frac{1+m}{2}nWef^{\prime \prime }(0)\right]f^{\prime \prime }(0),\frac{Nu_{x}}{\sqrt{Re}}=-\sqrt{1+m}\left(1+\frac{4}{3}Rd\right)\theta^{\prime }(0)$$

and $\frac{Sh_{x}}{\sqrt{Re}}=-\sqrt{1+m}\varphi^{\prime }(0)$, where $Re=\frac{U_{e}x}{\upsilon }$ is the local Reynold's number.

## 3 Method of solution

In this study, a powerful method called the homotopy analysis method (HAM), first developed in 1992 by Liao, has been implemented to obtain analytic approximations for the solution of the coupled nonlinear differential equations in Eqs. (8)-(10) together with the boundary conditions in Eqs. (11)-(12). Details of the method are available in [17]. Due to its efficiency, a number of authors employed the homotopy analysis method to solve nonlinear equations in their study works. For instance, the recent studies of Hayat et al. [18], Qayyum et al. [19] and Waqas et al. [20] show the successful application of the method to give convergent analytic approximations for the flow analysis of certain non-Newtonian fluid models.

In order to implement the homotopy analysis method in the study, we choose a set of basis functions in the form

(13) $$\left\{C_{m,n}\eta^{n}e^{-m\eta }:m\geq 0,n\geq 0\right\},$$

where $C_{m,n}$ are constant coefficients to be determined.

Then the auxiliary linear operators, denoted by $L_{f},L_{\theta }$ and $L_{\varphi }$, are selected in such a way that each solution of the homogeneous equations

(14) $$L_{f}[f(\eta )]=0,\;\;L_{\theta }[\theta (\eta )]=0,\;\;L_{\varphi }[\varphi (\eta )]=0$$

can be expressed as a linear combination of the base functions given in Eq. (13). More systematically, we construct each auxiliary linear operator by collecting high order linear terms of the corresponding operator whose detail is available in [21]. So, the following auxiliary linear operators are selected as:

(15) $$L_{f}=\frac{d^{3}f}{d\eta^{3}}+\frac{d^{2}f}{d\eta^{2}},\;\;\;L_{\theta }=\frac{d^{2}\theta }{d\eta^{2}}+\frac{d\theta }{d\eta },\;\;\;L_{\varphi }=\frac{d^{2}\varphi }{d\eta^{2}}+\frac{d\varphi }{d\eta },$$

satisfying the conditions $L_{f}[C_{1}+C_{2}\eta +C_{3}e^{-\eta }]=0,\;\;\;L_{\theta }[C_{4}+C_{5}e^{-\eta }]=0$, and $L_{\varphi }[C_{6}+C_{7}e^{-\eta }]=0$, where $C_{i}(i=1-7)$ are constants to be determined from the boundary conditions.

The corresponding initial approximations $f_{0}(\eta ),\;\theta_{0}(\eta )$ and $\varphi_{0}(\eta )$ are chosen in such a way that they agree with the solutions of the equations in Eq. (15). So, we choose the initial approximations in the form

(16) $$f_{0}(\eta )=C_{1}+C_{2}\eta +C_{3}e^{-\eta },\;\theta_{0}(\eta )=C_{4}+C_{5}e^{-\eta },\;\varphi_{0}(\eta )=C_{6}+C_{7}e^{-\eta }.$$

Enforcing the initial approximations to satisfy the given conditions Eqs. ((11)-(12)), the coefficients are determined and give

(17) $$f_{0}(\eta )=\delta -1+S+\eta +(1-\delta )e^{-\eta },\;\;\;\theta_{0}(\eta )=\frac{Bi_{1}}{1+Bi_{1}}e^{-\eta },\;\;\;\varphi_{0}(\eta )=\frac{Bi_{2}}{1+Bi_{2}}e^{-\eta }.$$

Finally, the auxiliary functions can also be selected as

$$H_{f}(\eta )=H_{\theta }(\eta )=H_{\varphi }(\eta )=e^{-\eta }.$$

To carry out the computation, we adopt the HAM-based Mathematica package, namely the BVPh 2.0 which was developed by Zhao and Liao [22]. The parameter values, $n=A=Da=Nb=Nt=0.1,\;\;We=0.2,\;\;\;m=1/3,\;\;Gr=0.5,\;\;Gc=0.4,\;\;M=0.1,\;\;Pr=1.0,\;\;\delta =0.3,\;\;Le=2,\;\;Q=Rd=0.3,\;\;Bi_{1}=Bi_{2}=0.5,\;\;S=0.3,\;\;\Omega =\pi /6$ and Ec=0.03, with 20th-order HAM approximations are considered throughout the manuscript unless otherwise stated.

**Figure 2:**
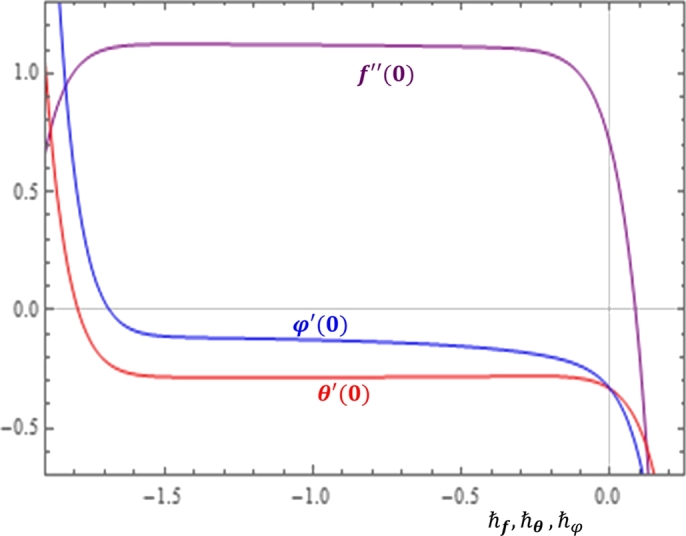
h-curves.

Despite the appropriate initial guesses, linear operators and auxiliary functions are selected, we still have a great freedom to take different values for the convergence-control parameters, $\hbar_{f},\;\hbar_{\theta }$ and $\hbar_{\varphi }$. Thus, proper selection of these parameters is required to get a convergent and accurate series solution. To do this, we plot the so-called *ℏ*-curves as shown in Fig. 2. It is shown in Fig. 2 that the *ℏ*-curves are nearly horizontal in the ranges

$$-1.7<\hbar_{f}<-0.3,\;\;\;-1.6<\hbar_{\theta }<-0.1\;\text{and}\;-1.5<\hbar_{\varphi }<-0.2.$$

According to Liao [17], these intervals are the valid regions in which taking any value of the parameters in the respective intervals can give us convergent solutions.

More systematically, the optimal value of each parameter can be determined by minimizing the average squared residual error

$$\varepsilon_{k}(\hbar )\approx \frac{1}{1+N}\sum_{j=0}^{N}{\left\{ℵ\left[\sum_{n=0}^{k}u_{n}(\eta_{j})\right]\right\}}^{2}.$$

Now, using the built functions of the BVPh 2.0 package, the optimal values for the convergence control parameters are:

$$\hbar_{f}\approx -1.23,\;\;\;\hbar_{\theta }\approx -0.87\;\;\;\text{and}\;\;\;\hbar_{\varphi }\approx -0.52.$$

Using these optimal values, we iterate the method to see the convergence of certain values of interest.

Table 1 displays that the values of the selected quantities of interest are convergent before the 30th order HAM and as the order of HAM increases, the squared residual errors are getting smaller.

**Table 1:** Convergence of HAM solutions.

Order of HAM				Squared residual errors		
Approximation	*f*″(0)	−*θ*′(0)	−*φ*′(0)	*ε*_*f*_	*ε*_*θ*_	*ε*_*φ*_
2	1.0798	0.2791	0.3478	3.5 × 10^−5^	6.9 × 10^−5^	2.2 × 10^−5^
6	1.0705	0.2804	0.3591	1.7 × 10^−6^	1.3 × 10^−5^	3.0 × 10^−6^
10	1.0698	0.2820	0.3587	3.2 × 10^−7^	3.7 × 10^−6^	1.7 × 10^−6^
14	1.0695	0.2828	0.3586	6.8 × 10^−8^	9.6 × 10^−7^	1.1 × 10^−6^
18	1.0693	0.2832	0.3586	1.6 × 10^−8^	3.2 × 10^−7^	6.2 × 10^−7^
22	1.0692	0.2834	0.3586	4.9 × 10^−9^	1.2 × 10^−7^	2.9 × 10^−7^
26	1.0691	0.2835	0.3586	2.1 × 10^−9^	6.1 × 10^−8^	1.4 × 10^−7^
30	1.0691	0.2835	0.3586	1.3 × 10^−9^	3.7 × 10^−8^	6.5 × 10^−8^

It is also possible to analyze the total error from the relation:

(18) $$\varepsilon_{k}^{t}=\varepsilon_{k}^{f}+\varepsilon_{k}^{\theta }+\varepsilon_{k}^{\varphi }$$

**Figure 3:**
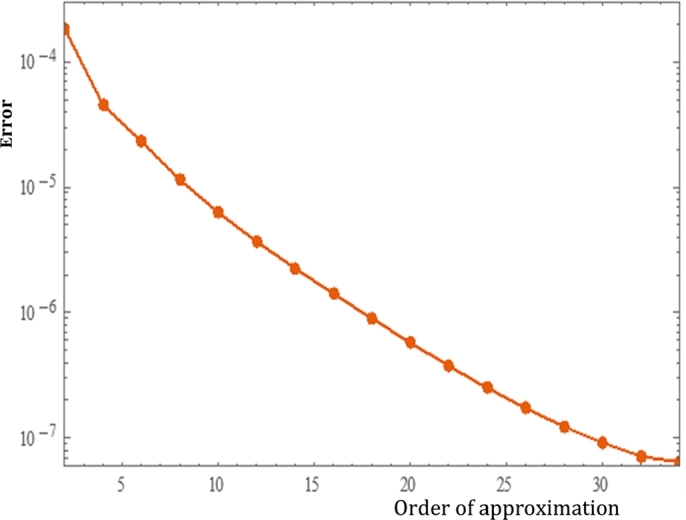
Total squared residual error.

The plot in Fig. 3 demonstrates the total squared residual error against some orders of HAM. The plot demonstrates that as the order of HAM approximation increases the total average squared residual error declines.

To further ensure the validity of our results, we make the following comparison of the present study with some of published results in the absence of the extended physical effects as depicted in Table 2.

**Table 2:** Comparison on values of *f*″(0) with the results of Khan and Pop [12] and Ullah and Zaman [23] (when *Pr* = 0.73, *Nb* = 0.0001, *Le* = 0.1, *Bi*_1_ → ∞, *Bi*_2_ → ∞, *n* = *δ* = *A* = *Da* = *M* = *Rd* = *Gr* = *Gc* = *S* = Ω = *Q* = *Ec* = 0) against some values of *m*.

m	Khan & Pop (2013)	Ullah & Zaman(2017)	Present study		
	*f*″(0)	*f*″(0)	*f*″(0)	−*θ*′(0)	−*φ*′(0)
0	0.4696	0.4696	0.4688	0.4228	0.2326
1/23	-	0.5690	0.5693	0.4598	0.2348
1/11	0.6550	0.6550	0.6554	0.4818	0.2453
1/7	-	0.7320	0.7322	0.5090	0.2516
1/5	0.8021	0.8021	0.8023	0.5402	0.2669
1/3	0.9277	0.9277	0.9277	0.5904	0.2699
1/2	1.0389	-	1.0389	0.6369	0.2822
1	1.2326	1.2326	1.2326	0.7202	0.3063
5	-	1.5504	1.5503	0.8648	0.3517
100	-	1.6794	1.6794	0.9252	0.3726
∞	-	1.6872	1.6872	0.9289	0.3734

Table 2 presents the comparisons of the values of $f^{\prime \prime }(0)$ against selected values of the wedge angle parameter *m*. It is observed that the values of $f^{\prime \prime }(0)$ obtained in the present study are in excellent agreement with the aforementioned published results. In Table 2, the coefficients of Nusselt number and Sherwood number of the present study are also presented.

## 4 Results and discussions

In this section, we present the outcomes of the study. Analysis is made to examine effects of the pertinent parameters on the dimensionless velocity $f^{\prime }(\eta )$, temperature θ(η) and nanoparticle concentration φ(η) profiles as well as on the coefficients of surface friction $f^{\prime \prime }(0)$, Nusselt number $\theta^{\prime }(0)$ and Sherwood number $\varphi^{\prime }(0)$ keeping other parameters fixed. The BVPh 2.0 is used to generate solutions graphically and numerically.

**Figure 4:**
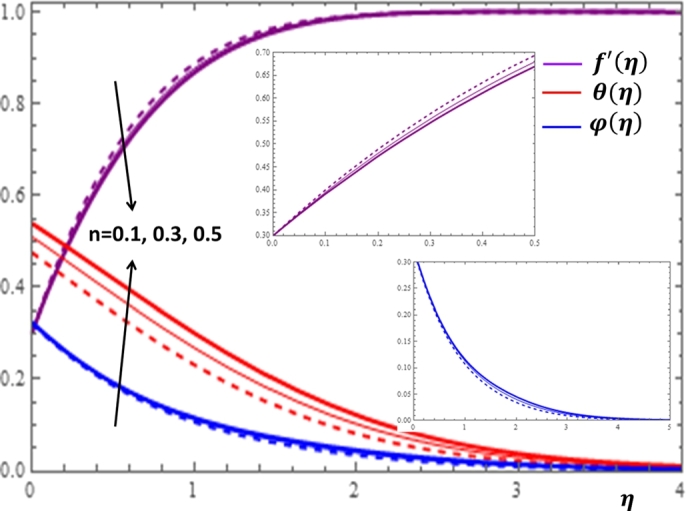
Impacts of the power law index *n* on *f*′(*η*), *θ*(*η*) and *φ*(*η*).

The behavior of the non-Newtonian nanofluid has considerable impacts on fluid velocity, temperature distribution and nanoparticles volume fraction in the boundary layer region. These effects can be expressed in terms of the power law fluid viscosity index *n* and its impacts on the flow field profiles are depicted in Fig. 4. It can be seen from Fig. 4 that the effect of *n* is more pronounced in upgrading the temperature profile. The increase in *n* also gives a gradual increment of the nanoparticle concentration in the boundary layer region. The velocity profile shows a decreasing behavior with the increase in the values of *n*. This holds because as the values of *n* increases, the nature of the fluid changes from shear thinning to shear thickening.

**Figure 5:**
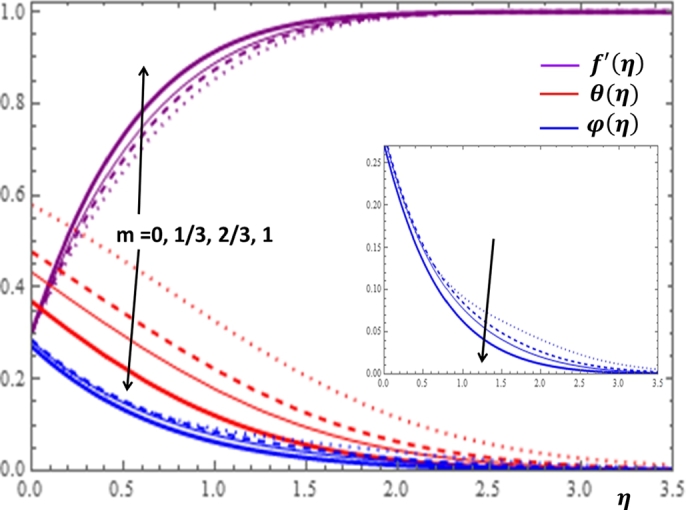
Impacts of the wedge angle parameter *m* on *f*′(*η*), *θ*(*η*) and *φ*(*η*).

In order to examine the influences of wedge angle on the dimensionless velocity, temperature and concentration profiles, it is worth mentioning that the Falkner-Skan power-law constant *m* defined as $m=\frac{\beta }{2-\beta }$ with *β* being the Hartree pressure gradient that can also be described in terms of the total wedge angle Ω by $\beta =\frac{\Omega }{\pi }$. Moreover, the value m=0 corresponds to β=0 or Ω=0 which implies that the wedge surface is horizontal. Similarly, m=1/3 corresponds to β=1/2 or $\Omega =\frac{\pi }{2}$. Further, m=1 corresponds to β=1 or Ω=π which implies that the wedge surface is vertical and the flow becomes a stagnation point flow. Thus, it is reasonable to examine the influence of the wedge angle in terms of the *m* as illustrated in Fig. 5. Fig. 5 illustrates that as the wedge angle increases, the fluid velocity is enhanced but the temperature and the nanoparticle volume fraction are declined. Physically, the increase in the wedge angle parameter corresponds to the increase in the applied pressure on the fluid. Further, the change in the values of *m* affect the temperature profile more significantly near the surface of the wedge.

**Figure 6:**
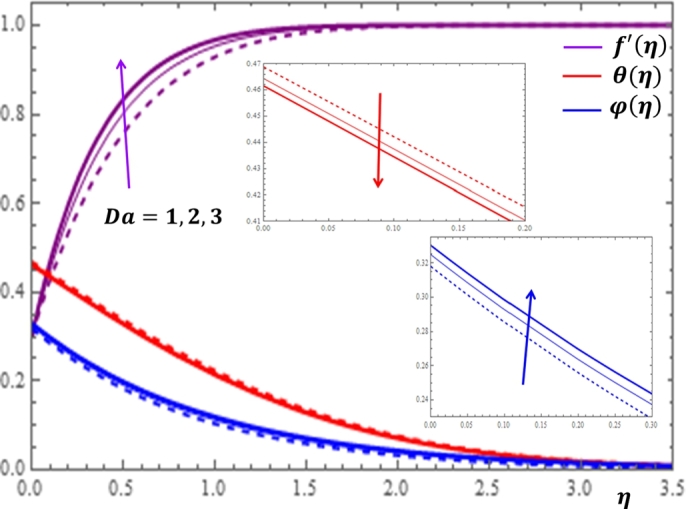
Impacts of Darcy number *Da* on *f*′(*η*), *θ*(*η*) and *φ*(*η*).

**Figure 7:**
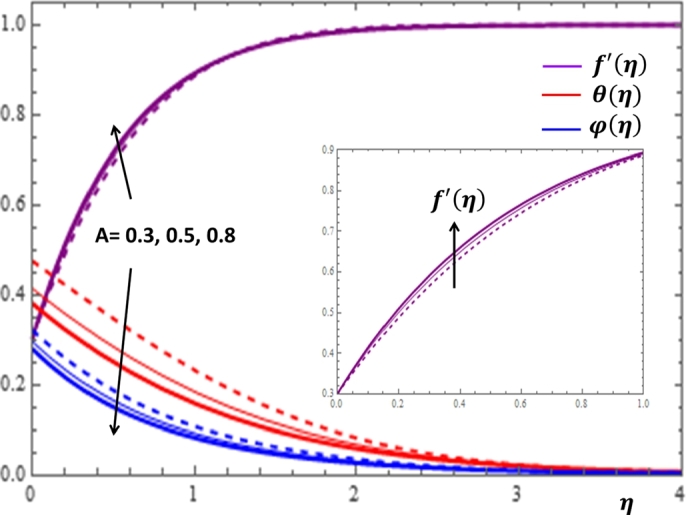
Impacts of the unsteadiness parameter on *f*′(*η*), *θ*(*η*) and *φ*(*η*).

The porosity of the medium is measured in terms of the Darcy number *Da* whose impact is more significant on the velocity profile as shown in Fig. 6. It is clearly observed in Fig. 6 that as the Darcy number *Da* increases, the velocity and nanoparticle concentration increase while the temperature declines slowly. This result is physically meaningful; because as the value of the Darcy number increases, permeability of the medium also increases which in turn minimizes the barriers placed along the flow path. This allows free flow of the nanofluid with enhanced velocity and concentration profiles in the boundary layer region. Fig. 7 displays that the increase in the unsteadiness parameter results the rise in the velocity profile but the decline in the temperature and concentration profiles.

**Figure 8:**
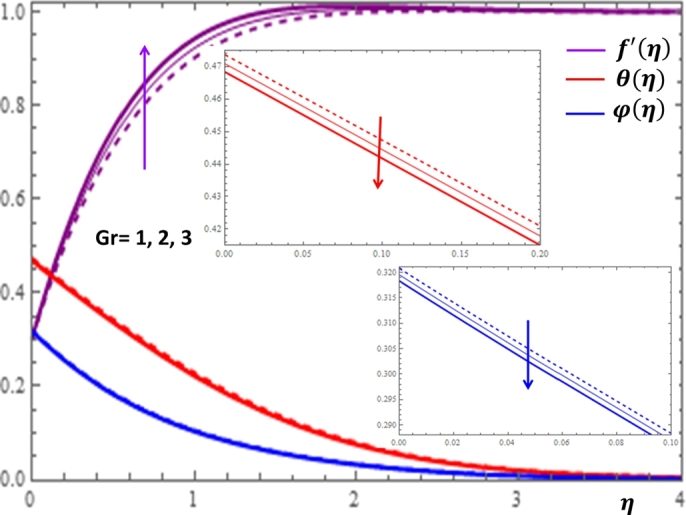
Impacts of thermal buoyancy parameter *Gr* on *f*′(*η*), *θ*(*η*) and *φ*(*η*).

**Figure 9:**
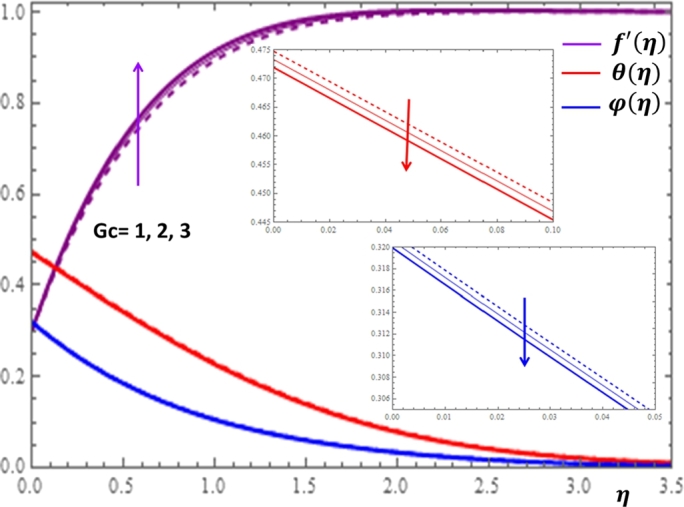
Impacts of concentration buoyancy parameter *Gc* on *f*′(*η*), *θ*(*η*) and *φ*(*η*).

**Figure 10:**
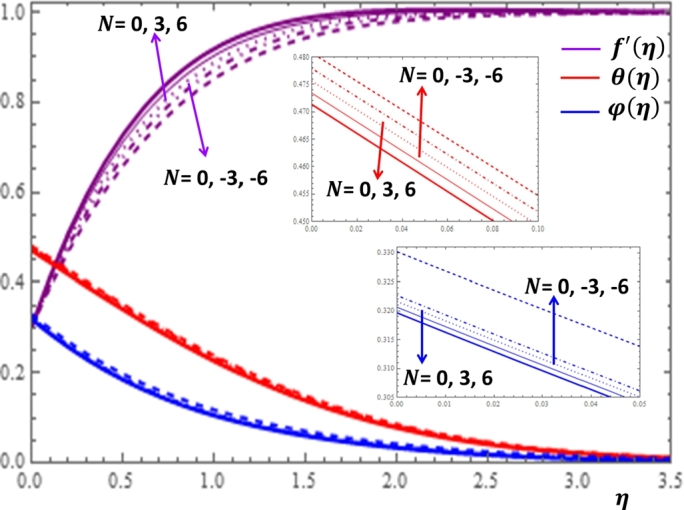
Impacts of buoyancy ratio parameter *N* on *f*′(*η*), *θ*(*η*) and *φ*(*η*).

The results given in Figs. 8 and 9 display the influences of thermal and concentration buoyancy parameters on $f^{\prime }(\eta )$, θ(η) and φ(η) profiles. It can be observed that the buoyancy parameters influence the velocity profile more significantly but no substantial variation is shown for the concentration profile with the changes in both parameters. The buoyancy force effect can also be expressed in terms of the buoyancy ratio parameter $N=\frac{Gc}{Gr}$ which is given in Fig. 10. Here, positive buoyancy force corresponds to assistive pressure gradient and negative buoyancy force acts like a resistive pressure gradient. It can be deduced from Fig. 10 that for assisting flow N>0, the velocity is increasing while the temperature and concentration are decreasing with the increase in the values of *N*. Opposite effects are shown for resistive flow N<0.

**Figure 11:**
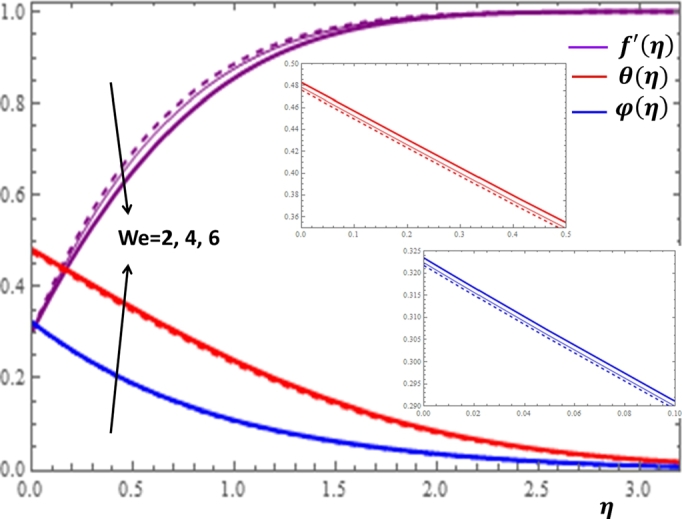
Impacts of Weissenberg number *We* on *f*′(*η*), *θ*(*η*) and *φ*(*η*).

The Weissenberg number *We* is defined as the ratio of fluid relaxation time to viscous forces. The impacts of *We* on $f^{\prime }(\eta )$, θ(η) and φ(η) are presented in Fig. 11. It can be noticed in Fig. 11 that as the values of *We* increases, the fluid velocity decreases while the temperature and concentration profiles gradually increase. This is true because the increase in *We* implies the increase in the relaxation time of the nanofluid or the thicker is the nanofluid which causes more resistance in the flow field.

**Figure 12:**
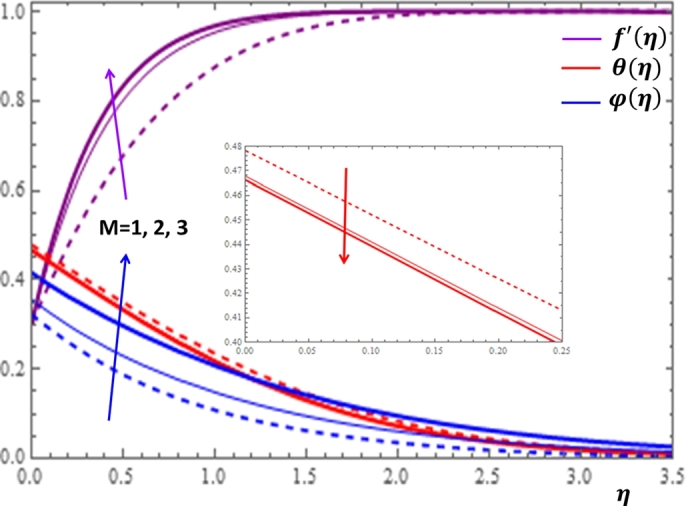
Impacts of magnetic field parameter *M* on *f*′(*η*), *θ*(*η*) and *φ*(*η*).

**Figure 13:**
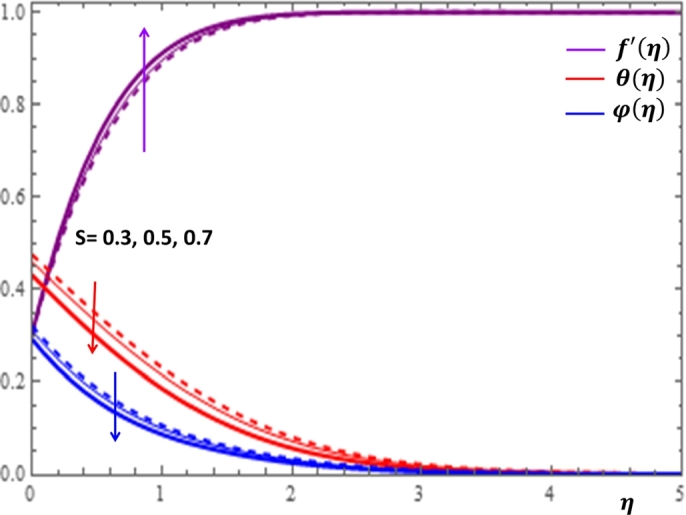
Impacts of injection parameter *S* > 0 on *f*′(*η*), *θ*(*η*) and *φ*(*η*).

**Figure 14:**
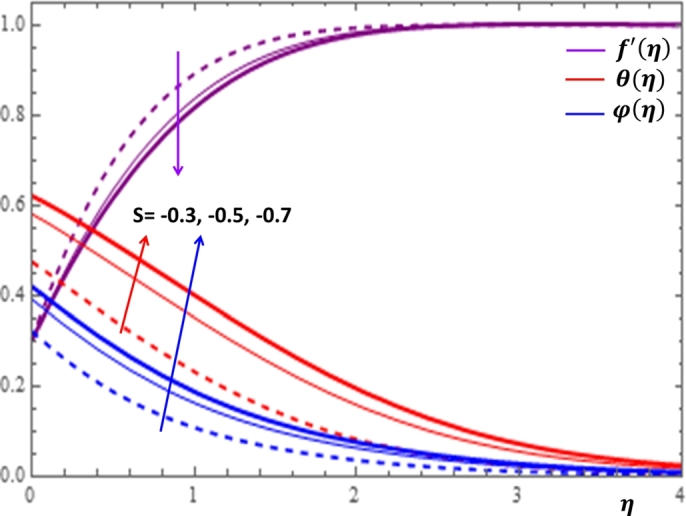
Impacts of suction parameter *S* < 0 on *f*′(*η*), *θ*(*η*) and *φ*(*η*).

**Figure 15:**
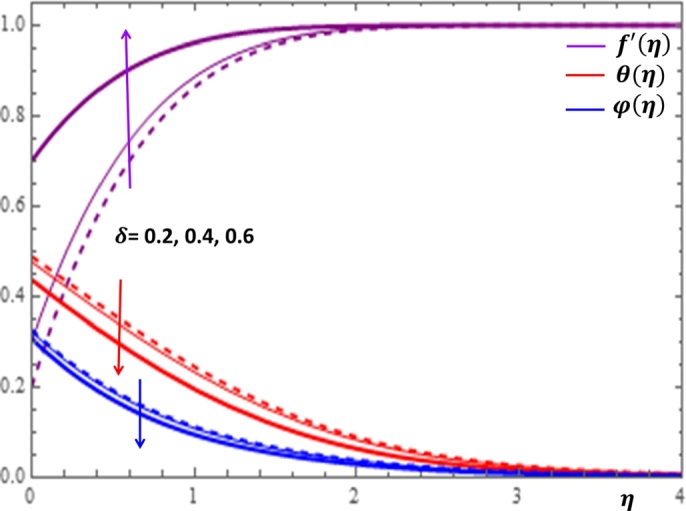
Impacts of velocity ratio *δ* (*δ* > 0) on *f*′(*η*), *θ*(*η*) and *φ*(*η*).

**Figure 16:**
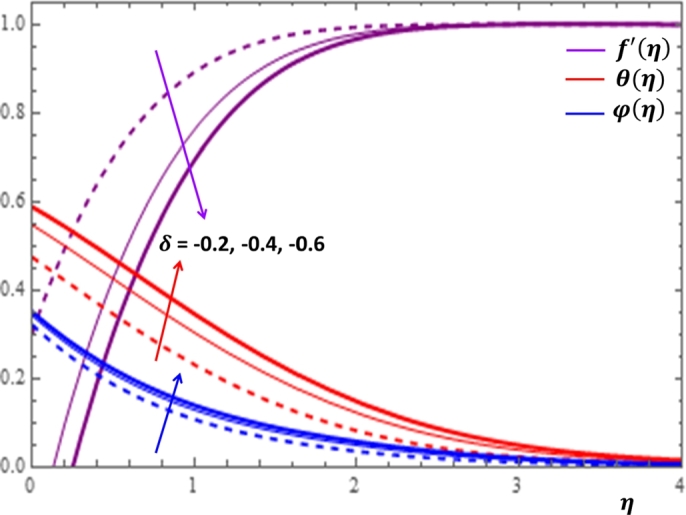
Impacts of shrinking velocity ratio *δ* on *f*′(*η*), *θ*(*η*) and *φ*(*η*).

**Figure 17:**
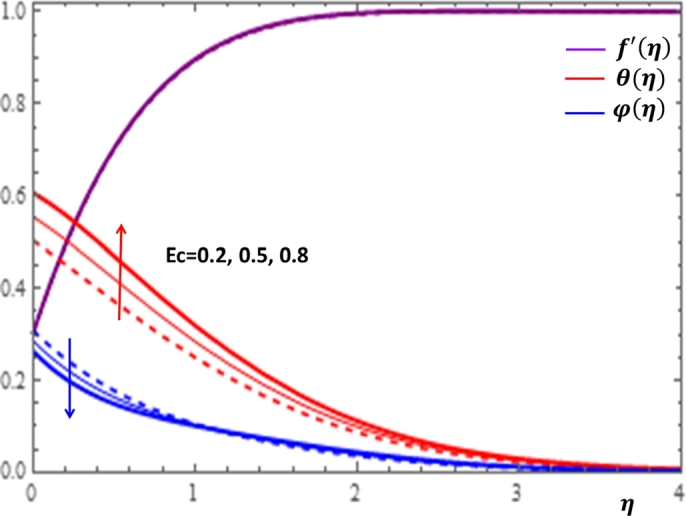
Impacts of the Eckert number *Ec* on *f*′(*η*), *θ*(*η*) and *φ*(*η*).

**Figure 18:**
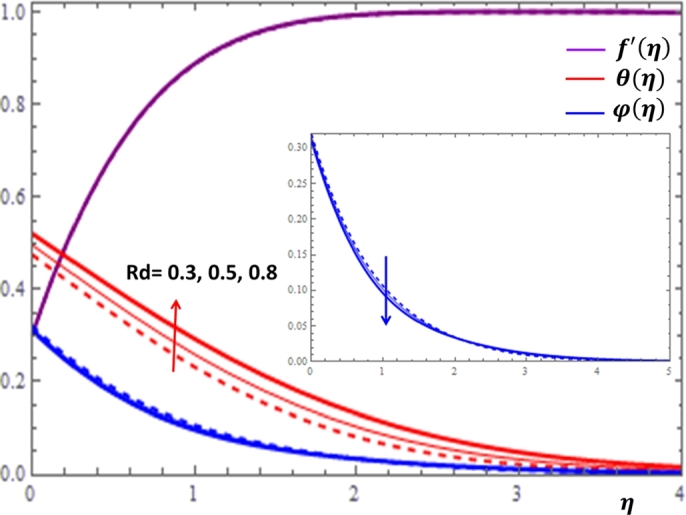
Impacts of thermal radiation *Rd* on *f*′(*η*), *θ*(*η*) and *φ*(*η*).

**Figure 19:**
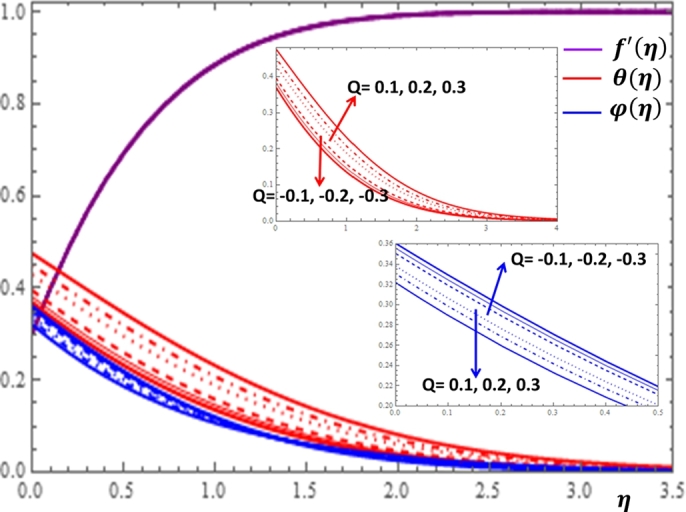
Impacts of heat source and sink *Q* on *f*′(*η*), *θ*(*η*) and *φ*(*η*).

The magnetic field effect expressed in the form $\frac{\sigma B_{0}^{2}}{\rho }(u-U_{e})$ can be viewed as the combination of the Lorentz force $\frac{\sigma B_{0}^{2}}{\rho }u$ and the imposed pressure $-\frac{\sigma B_{0}^{2}}{\rho }U_{e}$. It is clearly indicated in Fig. 12 that the increase in magnetic field strength leads to enhancement of the fluid velocity and concentration of the nanoparticles. However, the temperature profile decreases with the increase in magnetic field. The reason behind this observation is that for a forced convection, the external flow velocity $U_{e}$ is higher than the boundary layer fluid velocity *u*; that is, the imposed pressure dominates the Lorentz force. This effect plays the role of assisting force to facilitate the motion of the fluid and the removal of heat in the boundary layer region. Injection or suction of fluids through permeable walls play significant roles in many engineering and manufacturing activities such as in addition or removal of coolants or reactants in oil recovery, film cooling or coating of wires and fibers. Figs. 13 and 14 present the impacts of injection and suction parameters on the dimensionless velocity, temperature and concentration profiles. It is shown in Fig. 13 that as the injection parameter *S* increases, the fluid velocity rises where as the temperature and concentration profiles get declined. This is true because the warm fluid with the nanoparticles is taken away from the wedge surface. Opposite behavior is observed in the case of suction parameter as displayed in Fig. 14. Since the boundary layer flow is induced by the movement of both the wedge and the free stream flow of the nanofluid, the velocity ratio $\delta =\frac{U_{e}}{U_{w}}$ is used to measure the effects of such movements. The movement of the wedge occurs in the form of stretching ($U_{w}>0$) or shrinking ($U_{w}<0$) of its surface. Here, it is important to mansion that the value δ=0 defines the absence of free stream velocity ($U_{e}=0$) which can be described by the Sakiadis flat plate flow scenario; the value δ=1 corresponds to the Blasius flow situation in which the fluid and the wedge are moving with the same velocities. When the wedge is stretching (δ>0), the free stream and the wedge are moving in the same directions. In Fig. 15, one notices that as the velocity ratio for stretching wedge increases, the fluid velocity also increases; however, the temperature and concentration profiles are minimized. This holds as stretching of the wedge contributes as flow assistive role. Consequently, the velocity is enhanced while the temperature and concentration are declined with the increase in the velocity ratio parameter for the stretching wedge. Opposite behaviors can be seen in Fig. 16 when the wedge is shrinking (δ<0). As depicted in Fig. 17, a considerable effect of the Eckert number is seen on θ(η). The increase in the dissipation parameter *Ec* produces a frictional heating to be stored in the fluid. This enhances the temperature profile in the boundary layer. On the other hand, the concentration profile is minimized with the increase in *Ec* near the surface of the wedge. However, as we move far from the boundary layer region (η>1), the effect of imposed pressure dominates the effect of the dissipation parameter to slightly increase the nanoparticle concentration. No appreciable variation is observed for the velocity profile with respect to *Ec*. It is shown in Fig. 18 that the increase in *Rd* inspires significant enhancement of temperature in boundary layer region. This is as we expect in reality since the larger the thermal radiation corresponds to higher kinetic energy of the fluid particles. It is indicated in Fig. 19 that temperature and concentration profiles are found to be increasing functions of the heat source parameter; however, the profiles show opposite behaviors with the heat sink parameter. No considerable change is shown on velocity profile with the changes in both the heat source and heat sink parameters.

**Figure 20:**
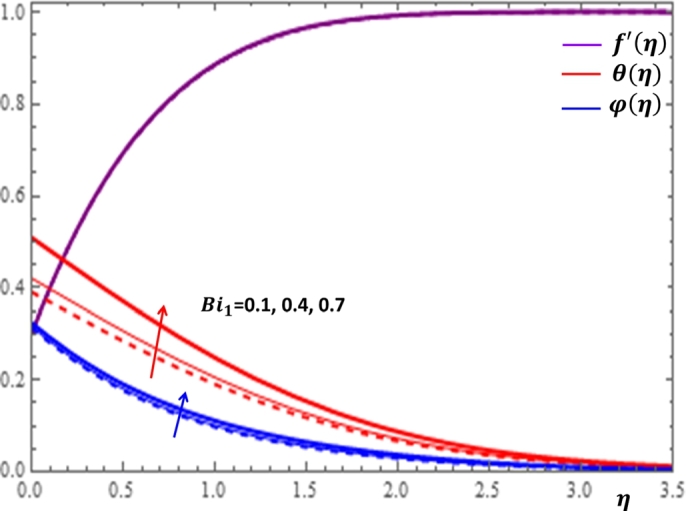
Impacts of Biot number for heat diffusion *Bi*_1_ on *f*′(*η*), *θ*(*η*) and *φ*(*η*).

**Figure 21:**
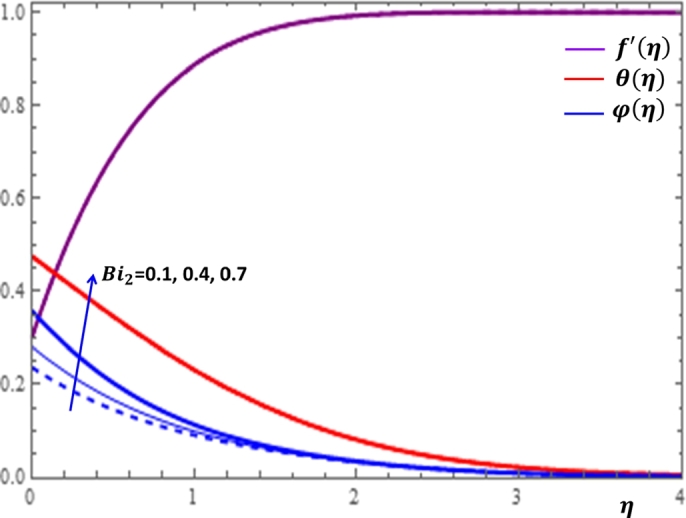
Impacts of Biot number for mass diffusion *Bi*_2_ on *f*′(*η*), *θ*(*η*) and *φ*(*η*).

The Biot number for heat diffusion is the ratio of convective heat transfer at the surface to conductive heat transfer within the flow region. The results on the variation of $f^{\prime }(\eta )$, θ(η) and φ(η) with respect to the Biot numbers for heat and mass diffusion are illustrated in Figs. 20 and 21. It is clear from Fig. 20 that there is a significant enhancement of temperature profile with the increase in the values of $Bi_{1}$. This is physically acceptable due to the fact that increasing Biot number provides stronger convection to produce higher temperature on the wedge surface. It is also shown in Fig. 21 that increasing the value of $Bi_{2}$ causes a considerable improvement in the concentration profile near the surface of the wedge and a relatively stable concentration distribution is observed after some point away from the wedge surface. Finally, we present some significant influences of the relevant parameters on the rates of momentum, heat and mass transfer processes in Table 3. It can be observed from Table 2 that the increases in the values of n,A and *Da* cause the enlargement in the coefficients of surface friction, Nusselt number and Sherwood number. On the other hand, the increase in the values of *We* and *m* leads to the reduction in the coefficients of skin friction, Nusselt number and Sherwood number.

**Table 3:** Impacts of Parameters on Coefficients of surface-friction, Nusselt number and Sherwood number.

n	A	Da	We	m	M	*δ*	Q	Rd	Ec	*f*″(0)	−*θ*′(0)	−*φ*′(0)
0.1										1.0828	0.2835	0.3587
0.2										1.1437	0.2848	0.3590
0.3	0.1									1.2135	0.2862	0.3594
	0.3									1.2655	0.3017	0.3652
	0.5	0.1								1.3170	0.3145	0.3706
		0.2								1.3557	0.3147	0.3708
		0.3	0.2							1.3931	0.3149	0.3709
			0.4							1.3378	0.3145	0.3708
			0.6	1/3						1.2914	0.3141	0.3707
				1/6						1.2636	0.3057	0.3701
				1/9	0.1					1.2517	0.3018	0.3699
					0.5					1.4053	0.3019	0.3708
					0.9	0.3				1.5386	0.3019	0.3714
						0.5				1.5386	0.3019	0.3714
						0.8	0.3			1.1571	0.3091	0.3728
							0.6			0.5267	0.2724	0.3859
							0.9	0.3		0.5463	0.1253	0.4158
								0.5		0.5473	0.1237	0.4140
								0.8	0.03	0.5478	0.1253	0.4116
									0.30	0.5485	0.1193	0.4131
									3.00	0.5548	0.0622	0.4264

## 5 Conclusions

In this study, a time-dependent, two-dimensional flow of tangent hyperbolic nanofluid towards a moving wedge is considered. Efforts have been made to examine the effects of various thermophysical effects by employing the homotopy analysis method, which is a relatively recent and powerful semi-analytic and semi-numerical method. The validity of the method as well as the BVPh2.0 package has been ensured by displaying the convergence of the solutions and accuracy of the findings as compared to previously published study results.

• Velocity is found to be facilitated by increasing the values of wedge angle parameter, porosity of the medium, assistive buoyancy force, magnetic field, injection and surface stretching parameters.
• The temperature distribution can be enhanced by increasing the power law index, suction, surface shrinking, Eckert number, thermal radiation, heat source and the Biot number for thermal diffusion.
• Concentration of nanoparticle can be maximized by increasing the values of Darcy number, magnetic field, suction, surface shrinking, heat sink and the Biot number for mass diffusion.
• The momentum, heat and mass transfer rates are found to be facilitated by the increase in the values of the power law index, unsteadiness parameter and permeability of the porous medium; and these rates can be slowed down by the increase in the values of Weissenberg number and wedge angle parameter. It is also shown that the increase in magnetic field, thermal radiation and dissipation parameters cause enhancements in momentum, heat and mass transfer rates, respectively.

## Declarations

### Author contribution statement

Tesfaye Kebede: Conceived and designed the experiments.

Eshetu Haile: Analyzed and interpreted the data.

Gurju Awgichew: Contributed reagents, materials, analysis tools or data.

Tadesse Walelign: Performed the experiments; Wrote the paper.

### Funding statement

This research did not receive any specific grant from funding agencies in the public, commercial, or not-for-profit sectors.

### Competing interest statement

The authors declare no conflict of interest.

### Additional information

No additional information is available for this paper.
